# The Gαi protein subclass selectivity to the dopamine D_2_ receptor is also decided by their location at the cell membrane

**DOI:** 10.1186/s12964-020-00685-9

**Published:** 2020-12-11

**Authors:** Agnieszka Polit, Beata Rysiewicz, Paweł Mystek, Ewa Błasiak, Marta Dziedzicka-Wasylewska

**Affiliations:** Department of Physical Biochemistry, Faculty of Biochemistry Biophysics and Biotechnology, Jagiellonian University, Gronostajowa 7, 30-387 Kraków, Poland

**Keywords:** Heterotrimeric G proteins, Dopamine D2 receptor, Functional selectivity, Protein–membrane interaction, FLIM–FRET, FRAP

## Abstract

**Background:**

G protein-coupled receptor (GPCR) signaling via heterotrimeric G proteins plays an important role in the cellular regulation of responses to external stimuli. Despite intensive structural research, the mechanism underlying the receptor–G protein coupling of closely related subtypes of Gαi remains unclear. In addition to the structural changes of interacting proteins, the interactions between lipids and proteins seem to be crucial in GPCR-dependent cell signaling due to their functional organization in specific membrane domains. In previous works, we found that Gαs and Gαi_3_ subunits prefer distinct types of membrane-anchor lipid domains that also modulate the G protein trimer localization. In the present study, we investigated the functional selectivity of dopamine D_2_ long receptor isoform (D_2_R) toward the Gαi_1_, Gαi_2_, and Gαi_3_ subunits, and analyzed whether the organization of Gαi heterotrimers at the plasma membrane affects the signal transduction.

**Methods:**

We characterized the lateral diffusion and the receptor–G protein spatial distribution in living cells using two assays: fluorescence recovery after photobleaching microscopy and fluorescence resonance energy transfer detected by fluorescence-lifetime imaging microscopy. Depending on distribution of data differences between Gα subunits were investigated using parametric approach–unpaired *T*-test or nonparametric–Mann–Whitney *U* test.

**Results:**

Despite the similarities between the examined subunits, the experiments conducted in the study revealed a significantly faster lateral diffusion of the Gαi_2_ subunit and the singular distribution of the Gαi_1_ subunit in the plasma membrane. The cell membrane partitioning of distinct Gαi heterotrimers with dopamine receptor correlated very well with the efficiency of D_2_R-mediated inhibition the formation of cAMP.

**Conclusions:**

This study showed that even closely related subunits of Gαi differ in their membrane-trafficking properties that impact on their signaling. The interactions between lipids and proteins seem to be crucial in GPCR-dependent cell signaling due to their functional organization in specific membrane domains, and should therefore be taken into account as one of the selectivity determinants of G protein coupling.

**Video abstract**

## Background

Dopamine D_2_ receptor (D_2_R) is one of the class A G-protein-coupled receptors (GPCRs), whose interaction with the heterotrimeric GTP-binding proteins (or G proteins) induces various cellular responses. GPCR proteins are the most recognized target by about 35% of the available drugs [1]. For example, the classical drugs used in the treatment of schizophrenia or Parkinson’s disease represent the ligands of dopamine receptors. Based on the cellular response induced by their activation, dopamine receptors are divided into two groups: D1-like (D_1_, D_5_) and D2-like (D_2_, D_3_, D_4_). The D_2_R long isoform, which is studied in the present work, is a member of the D2-like group. The characteristic feature of this group is the inhibition of adenylyl cyclase, which leads to a decrease in the level of cAMP via interaction with the Gαi/o class of G proteins. By contrast, the D1-like group induces the opposite effect on the level of cAMP by interacting with the Gαs subunits, which in turn activates adenylyl cyclase [2, 3].

Depending on the activity of the Gα subunit, the most recognized partners of GPCRs —heterotrimeric G proteins—are divided into four classes as follows: Gi/o, Gs, Gq, and G_12/13_. The heterotrimeric G proteins consist of three components—α, β, and γ—forming a trimer in an inactive state, which binds the GDP nucleotide. After activation by a receptor, the bound GDP nucleotide is exchanged for GTP, triggering the dissociation of the trimer into a Gα subunit and a Gβγ dimer; both these components induce various downstream effects leading to many cellular responses. The Gα subunit is composed of two domains: a GTPase domain, which is responsible for autoregulation via GTP hydrolysis; and a helical domain, which interacts with partners such as the RGS proteins (regulator of G protein signaling), effectors, and the Gβγ dimer. Changes in the conformation of the helical domain are also implicated in the enzymatic cycle and the overall activity of the G proteins [4]. It has been identified that N-terminus is the site of lipidation which enables docking at the surface of the lipid bilayer, while Gγ prenylation influences the plasma membrane localization of the G proteins. Furthermore, lipid modifications of the Gα subunit differ among various classes of G proteins. In the case of Gαi, N-myristylation and S-palmitoylation occur, whereas in Gαs N- and S-palmitoylation take place. N-acylation of the lipid moiety is irreversible, but it may be insufficient to allow stable docking at the surface of the lipid bilayer [5]. The second reversible modification occurring at the cysteine residue is proposed as one of the mechanisms that regulate the localization and performance of the G proteins. It has been postulated that activation triggered deprivation of cysteine modification can lead to depletion or enrichment of the protein population at different stages of signal transduction [6–10].

Gi/o class consists of two subclasses: Gαi and Gαo. The Gαi subclass is composed of Gαi_1_, Gαi_2_, and Gαi_3_ (genes GNAI1, GNAI2, GNAI3), and the Gαo subclass is composed of Gαo_1_ and Gαo_2_ (genes GNAO1, GNAO2). These proteins show profound homology (approximately 70% amino acid sequence identity) but vary in other features such as electrostatic properties [11]. While D_2_R is expressed mostly in the basal ganglia (as well as in other brain regions such as the midbrain, thalamus, hypothalamus, and cerebral cortex), none of the three Gαi subunits show any regional specificity in the brain and are present in the regions where D_2_R is expressed. However, the mRNA levels of these subunits vary—Gαi_2_ has a similar prevalence as Gαo_1_—whereas the levels of Gαi_1_ and Gαi_3_ are relatively lower [12].

The following three regions of the Gα subunit are identified to be involved in the interaction with receptor: C-terminal helix, α4–β6 loop, and to a lesser extent, αN–β1 loop [13, 14]. The last six amino acids in the C-terminus appear to have the most profound impact as a determinant of selectivity in the G protein–receptor interaction [15, 16]. However, the role of the C-terminus of Gα in interactions with receptors is heterogeneous among GPCRs. In particular, in the case of receptors interacting with Gαi, the other regions of this subunit are thought to reduce the impact of its C-terminus in the interaction with the receptor (the Gαi subunits show high similarity in the C-terminal residues, and only Gαi_3_ differs in the identity of amino acids in two positions). Furthermore, in contrast to other GPCRs, the Gαi-interacting receptors exhibit selectivity toward specific Gα subunits to a greater extent [17]. On the other hand, the second and third intracellular loops (2ICL, 3ICL), together with the transmembrane helix (TM)—TM3, TM5, and TM6, are recognized as the most relevant regions of GPCRs in terms of their interaction with suitable G proteins [18]. Even closely related receptors show differences in the secondary structure of these regions. Changes in the secondary structure of 2ICL and the length of 3ICL are proposed to influence the selectivity toward G proteins [19]. Nevertheless, in the case of both G proteins and GPCRs, these determinants are not fully recognized yet. In addition to the C-terminus of Gα subunit which acts as a determinant of selectivity of receptor–G protein coupling, there exist other determinants (although not so well documented) that are equally, and sometimes even more important in the recognition of G protein by many receptors [16, 17, 20, 21]. The following factors may affect this process: ligand used to stimulate the receptor, time of stimulation, interacting partners (such as RGS, AGS (receptor-independent activators of G protein signaling), and others), receptors oligomerization, and the lipid composition of the cell membrane [15, 22–24].

Dopamine D_2_R is capable of coupling more than one G protein while modulating the formation of cyclic AMP. The ability to inhibit the activity of adenylate cyclase depends on the ability to couple one, or more, of the Gi/o subunits [25–27]. However, the mechanism by which the receptor can selectively discriminate between the closely related subtypes of G proteins and involvement of other factors still remains unclear. It has been shown earlier that dopamine D_2_R may differentially couple the Gαi and Gαo subtypes in a receptor agonist-dependent manner, leading to diverse functional outcomes [25, 27]. However, most of these interactions were analyzed using a system that measures intracellular events (e.g. cAMP accumulation, calcium mobilization) or in isolated membrane fractions (e.g. radioligand binding studies). Since the Gαi and Gαo proteins are closely related, it is further difficult to separate their signaling when working on isolated membrane fractions. Most of the studies exploring the role of protein–protein interactions neglect the interactions of the signaling proteins with lipids, as well as the participation of the lipid bilayer itself in the processes of signal transduction. One of the important aspects that have not been fully explored is the impact of the plasma membrane on the efficiency and selectivity of the G proteins signaling. The mutual influence of lipids and membrane proteins along with cytoskeleton is considered as a factor that may promote their nanoclustering and organization into dynamic signaling platforms [28, 29].

Similarly, in the case of trimeric G proteins, partitioning occurs in different regions of the cell membrane. A review of the published data indicated that the Gαi proteins reside in the ordered parts of the membrane that are rich in cholesterol and sphingolipids [30, 31]. It is assumed that the partitioning process is driven not only by the lipid moieties attached to proteins but also by interaction with other components residing in such clusters (i.e. caveolins) [31, 32]. Moreover, the specific membrane targeting of G proteins is affected by the Gβγ dimer which seems to determine the preference toward the less-ordered segments of the lipid bilayer [31, 33, 34]. These observations indicate that localization may change in different states of the signal transduction process when the G protein trimer dissociates or associates. In addition, it has been postulated that ligand binding induces changes in the localization of GPCRs [35, 36]. Regardless of the signals within proteins, such as palmitoylation which is a signal stated to localize in the ordered membrane regions [37], other factors may also influence the overall outcome in the compartmentalization process.

In the present study, we analyzed the behavior of the three closely related Gαi proteins and dopamine D_2_R in a lipid bilayer environment in the context of activation selectivity. We monitored the dynamics and the mutual proximity of D_2_R and Gαi_1_, Gαi_2_, or Gαi_3_, as well as their heterotrimers formed with the Gβ_1_γ_2_ dimer, using two highly selective and sensitive assays: fluorescence resonance energy transfer (FRET) and fluorescence recovery after photobleaching (FRAP). These approaches allowed comparing the receptor and G proteins directly at the living cell membrane in their native dynamic environment, without relying on downstream signals such as the production of second messengers. Surprisingly, although the Gαi proteins showed high similarity, our results revealed significant differences not only in their rate of lateral diffusion within the plasma membrane but also in their colocalization with dopamine D_2_R. We found that the cell membrane partitioning of particular Gαi heterotrimers and dopamine D_2_R showed a good correlation with the efficiency of D_2_R-mediated inhibition of cAMP. These results suggest that the membrane distribution of signaling partners can be investigated in depth in terms of how it contributes to the selectivity of the G protein–receptor coupling. To the best of our knowledge, this is the first report to show that the Gαi subunits differ in their membrane-trafficking properties that impact on their signaling, as the membrane localization of the Gαi_1_, Gαi_2_, and Gαi_3_ subunits has been considered to be identical so far.

## Methods

### Site-directed mutagenesis

All the genes encoding human Gα subunits (GNAI1, GNAI2, GNAI3, GNAS) and dopamine D_2_R long isoform (DRD2L) were purchased from UMR cDNA Resource Center (Bloomsburg, PA, USA), and the sequences of fluorescent proteins (FP) were obtained from Clontech (Mountain View, CA, USA).

The sequences coding for mCitrine or mGFP were inserted into the αb–αc loop of the human Gαi subunits through Overlap Extension PCR Cloning [38]. In the case of Gαi_1_ and Gαi_2_, the sequences were inserted after Ala121, while for Gαi_3_, they were inserted after Ala114 [39]. The sequence of FP was flanked by Ser–Gly and Gly–Ser linkers. The mCitrine and mGFP sequences were obtained as described previously [40]. In the Gαs subunit, the FP sequence was incorporated between the helical and GTPase domains as described previously [40]. The sequences of all the Gα subunit fusion proteins were obtained in a pcDNA3.1+ vector (Invitrogen, Thermo Fisher Scientific, Inc., Waltham, MA, USA). The D_2_R-mCherry construct (with mCherry fused to the C-terminus of D_2_R) was prepared by introducing restriction sites to DRD2 through polymerase chain reaction and then by cloning the dopamine receptor gene into the pmCherry-N1 vector (Clontech) using NheI and XhoI enzymes. Additionally, to ensure the correct location of the described fusion protein in the membrane, it was necessary to extend the linker between the proteins, which was achieved using a 35-amino acid linker with a flexible character consisting of repeated GGSG sequences.

### Cell culture and transfection

The human embryonic kidney 293 cells (HEK293) (ATCC, Manassas, VA, USA) were cultured in Minimum Essential Medium (MEM) (Thermo Fisher Scientific, Inc., Waltham, MA, USA) with 10% fetal bovine serum (FBS) (Sigma Aldrich, Poznań, Poland) under 5% CO_2_ at 37 °C. For imaging experiments, the cells were seeded onto sterile glass coverslips and cultured in 30-mm plates, while for determining the levels of cAMP, the cells were seeded onto six-well plates coated with 0.5% gelatin (Type A; BioShop Canada Inc., Montréal, Canada). Transient transfection was performed using the TransIT-X2® Dynamic Delivery System (Mirus Bio, Madison, WI, USA) according to the manufacturer’s instruction. The amounts of DNA used for each experiment were as follows: determination of cAMP levels—0.9 or 1.7 μg DNA per well; FLIM–FRET and FRAP—0.1–0.45 μg DNA per dish. The ratio of DNA (Gα-D_2_R) used was as follows: determination of cAMP levels: 1–1.25; FLIM–FRET and FRAP: 1:1.5; in case of overexpression of trimer, Gβ, Gγ and Gα were used in equimolar DNA amounts. All the experiments were performed 2 or 3 days after transfection.

### Live-cell imaging microscopy

Leica SP5 II SMD confocal microscope (Leica Microsystems, Mannheim, Germany) or Leica TCS SP5 confocal scanning microscope (Leica Microsystems, Mannheim, Germany) with a 63 × 1.4 numerical aperture and a oil-corrected objective lens was used for the observation of cells. Fluorescence of mCitrine or mGFP was acquired at 495–570 nm with an excitation wavelength of 488 nm (argon ion laser), and that of mCherry at 610–700 nm with an excitation wavelength of 594 nm (laser diode). During observation, the cells were kept at 37 °C in an air–steam cube incubator in Dulbecco’s Modified Eagle Medium (DMEM-F12; without phenol red) (Thermo Fisher Scientific, Inc., Waltham, MA, USA) supplemented with 2% FBS.

### cAMP level measurements

The concentration of cAMP was determined in cell lysates using cAMP ELISA chemiluminescence kit (STA-500; Cell Biolabs Inc., San Diego, CA, USA). Three days after transfection, the HEK293 cells were stimulated with 1 µM rotigotine hydrochloride (Sigma Aldrich, Poznań, Poland), a D_2_R agonist, for 10 min. Prior to stimulation, the cells transfected with Gαi were incubated in a medium containing 1 µM forskolin (Sigma Aldrich, Poznań, Poland) for 5 min. These prestimulation and stimulation procedures were conducted in MEM supplemented with 0.5% FBS. After stimulation, the cells were harvested and their cAMP concentration was determined according to the manufacturer’s instructions. In each case, four independent experiments were performed in duplicates. Nontransfected cells were used as controls, and the concentrations of cAMP in transfected cells were normalized in comparison with the values determined in controls in each experiment.

### FLIM–FRET measurements

The cells were observed using Leica SP5 II SMD confocal microscope with an integrated module PicoHarp 300 Time-Correlated Single Photon Counting (TCSPC) system (PicoQuant, Berlin, Germany). The experiments were conducted as described earlier in detail [34]. Confocal images of the cells were collected prior to each FLIM measurement. mCitrine (energy donor) and mCherry (energy acceptor) were used as the FRET pair. The FLIM–FRET experiments were carried out on live HEK293 cells expressing appropriate levels of Gα-mCitrine (donor alone: Gα-mCitrine or Gα-mCitrine with Gβ_1_γ_2_) and D_2_R-mCherry (donor and acceptor: Gα-mCitrine with or without Gβ_1_γ_2_ and D_2_R-mCherry). Excitation was performed using a pulsed laser diode (Leica; 40 MHz) at 470 nm. Emission from 500 to 550 nm was collected with an avalanche photodiode using a fluorescence band-pass filter. All the images were recorded in 512 × 512 format with an acquisition time of approximately 3–4 min. In each experiment, the cells with only donor and those with donor–acceptor were observed, and the level of fluorescence of mCitrine and mCherry was estimated. In the case of cells treated with rotigotine (1 μM) for the stimulation of D_2_R, the ligand was added immediately after the imaging was started and the images were collected for up to 15 min after stimulation.

To quantify the apparent fluorescence lifetimes in the plasma membrane, we manually selected the regions of cell areas in each image and fitted the fluorescence lifetime histograms with double-exponential decay functions using SymPhoTime software (PicoQuant, Berlin, Germany). In the case of each image, the FRET efficiency was calculated for the FRETing state with the equation: $E = 1 - \left( {\tau_{DA} /\tau_{D} } \right)$ by comparing the donor lifetimes in the presence (*τ*_*DA*_) and absence (*τ*_*D*_) of the acceptor [41].

### FRAP measurements

All the FRAP experiments were performed and results were analyzed as described earlier [40]. As high photostability was required, mGFP-tagged fusion proteins of Gα subunits were used in these experiments. Briefly, the transiently transfected live HEK293 cells were incubated at 37 °C. Just before imaging, the culture medium was replaced with fresh DMEM-F12 medium enriched with 2% FBS. The FRAP images were collected for at least 100 s after the photobleaching impulse using Leica TCS SP5 confocal scanning microscope equipped with LAS AF software and a 63 × 1.4 NA oil-immersion lens.

### Statistical analysis

Data distribution was determined using Shapiro–Wilk *W* test and skewness and kurtosis analysis. Depending on the approach applied (unpaired *T*-test for parametric data and Mann–Whitney *U* test for nonparametric data), the results are presented as mean ± standard error of the mean (SEM) or median ± median absolute deviation (MAD). The details of the statistical analysis were described previously [34].

## Results

### Functionality of created fusion proteins

The purpose of this work was to investigate the differences in the coupling selectivity of the three Gαi subunits of G proteins (Gαi_1_, Gαi_2_, Gαi_3_) toward dopamine D_2_R in living cells. Their mutual colocalization was observed in basal conditions without receptor stimulation and after stimulation with a full agonist—rotigotine. Two approaches were used for analyzing the proteins of interest: FLIM–FRET and FRAP measurements with the use of FPs (mCitrine, mGFP, or mCherry) as tags. Such approaches require more attention during the creation of fusion proteins. In addition, the incorporation of FPs may have a profound impact on the conformation of the investigated proteins and may also influence their functionality, localization and at the tail end—expression level. To address this last uncertainty, we examined the levels of mRNA of all Gα-FP fusion proteins used in co-expression with dopamine D_2_ receptor with or without company of Gβγ subunits. We saw, that the relative expression of studied proteins remained constant in each experimental set-up (Additional file 1).

**Fig. 1:**
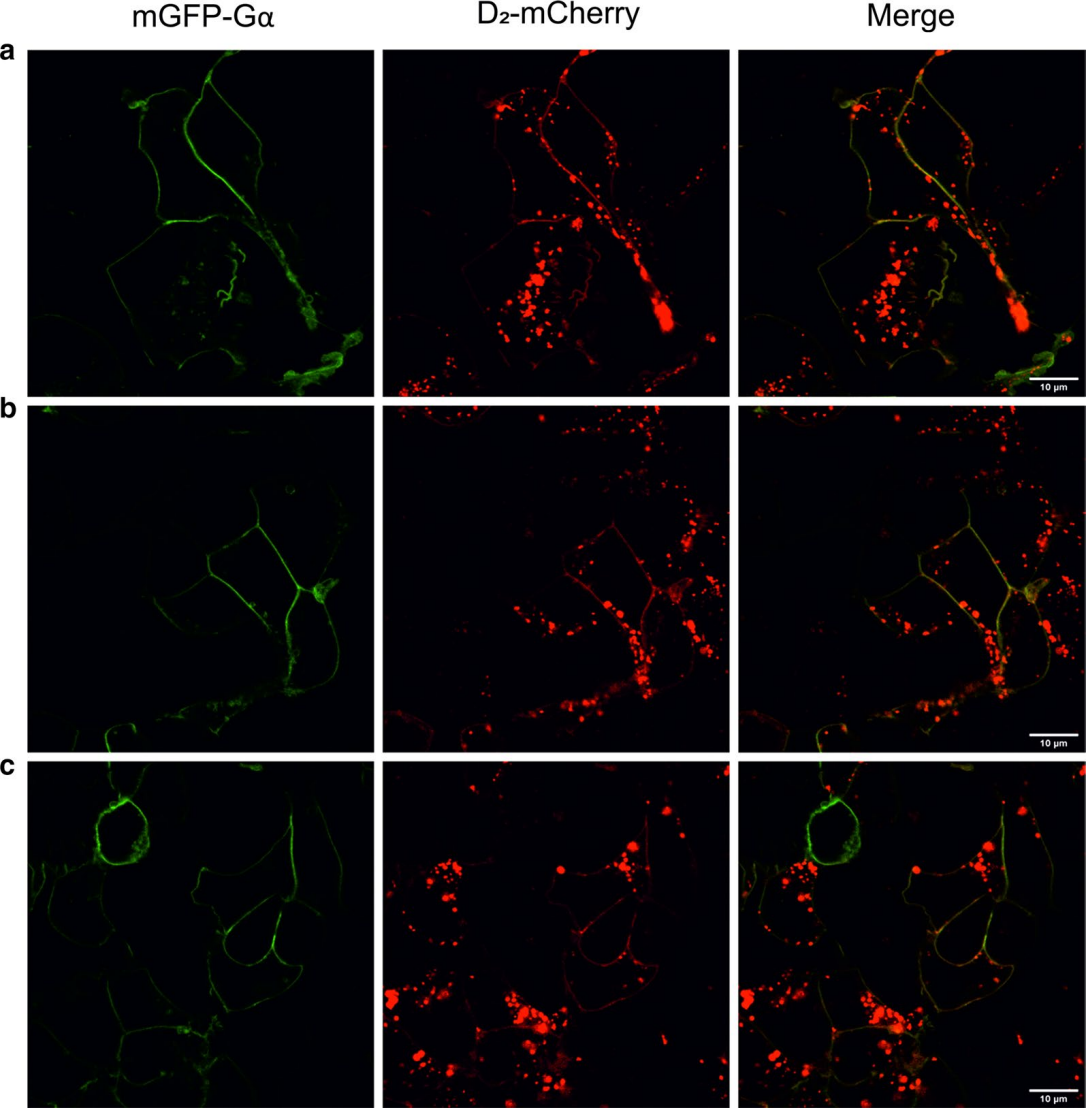
Cellular localization of Gαi subunit heterotrimers with dopamine D_2_ receptor. Representative confocal images of FP-tagged Gαi-mGFP subunits with Gβ_1_γ_2_ dimer and D_2_R-mCherry receptor in transiently cotransfected HEK293 cells. Localization of the investigated proteins: **a** Gαi_1_β_1_γ_2_-D_2_R, **b** Gαi_2_β_1_γ_2_-D_2_R, and **c** Gαi_3_β_1_γ_2_-D_2_R. Scale bar, 10 µm

In the case of Gαi proteins, mCitrine or mGFP was incorporated into the second loop (αb–αc) after A114 (Gαi_3_) or A121 (Gαi_1_, Gαi_2_), flanked with short linkers, based on the results reported by Gibson and Gilman for Gαi_3_ [34, 39]. This process minimizes the possibility of disruption of the interaction between the Gα subunit and D_2_R (via C-terminus of Gα) and the effect on their localization at the surface of the cell membrane which occurs via the N-terminus. All proteins were properly localized at the cell membrane; this is especially noticeable in the case of overexpression of the complete trimer (Fig. 1). Gαs, investigated as subunit non-interacting with D_2_R as well as with different characteristics, also exhibited proper cellular localization and a similar behavior as Gαi with reference to the influence of Gβ_1_γ_2_ on localization. The Gαs subunit was fused with mCitrine or mGFP cloned between the helical and GTPase domains by replacing amino acids 72–82 with an FP sequence and adding short linkers [42].

**Fig. 2:**
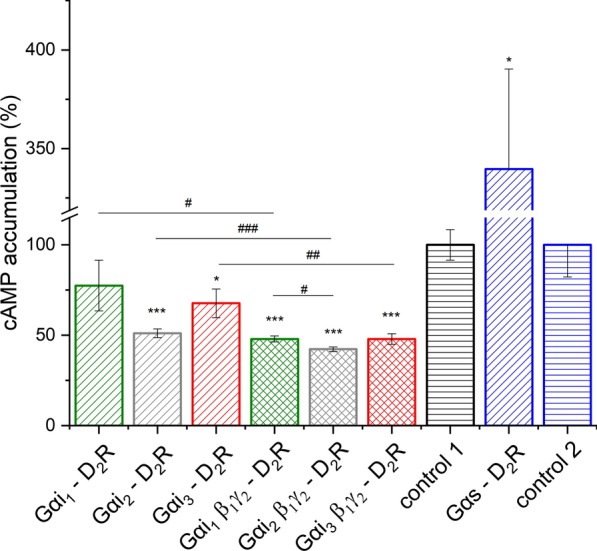
Changes in the intracellular cAMP level after stimulation of dopamine D_2_ receptor with rotigotine. HEK293 cells transiently transfected with Gα-mCitrine and D_2_R-mCherry or Gα-mCitrineβ_1_γ_2_ and D_2_R-mCherry were prestimulated with 1 μM of forskolin to enhance the basal levels of cAMP. After 5 min, rotigotine was added at a final concentration of 1 μM and the cells were incubated with the agonist for 10 min. In the case of Gαs, the prestimulation step with the use of forskolin was omitted. After stimulation, the cells were harvested and their intracellular cAMP concentration was determined. Data are presented as percentage of the cAMP levels in controls (nontransfected cells), and an appropriate control was used in every experiment. Control 1—Gαi; Control 2—Gαs. Error bars represent SEM; n = 4 experiments were performed in duplicates; unpaired *T*-test was used to evaluate the differences between samples. Comparison with adequate control: *p < 0.05 and ***p < 0.001; comparison within Gαi: ^#^*p* < 0.05, ^##^*p* < 0.005, and ^###^*p* < 0.001

The functionality of some of the G proteins prepared in this manner was already verified by other authors. They found that these insertions did not change the properties of the proteins, such as their interaction with adenylyl cyclase, localization at the surface of the cell membrane, or the process of nucleotides exchange [39, 42]. Nevertheless, the activity of all the proteins was investigated by taking into account their ability to inhibit adenylyl cyclase after the activation of D_2_R. Our previous studies have indicated that the differences in the response of Gαi_3_-mCitrine or Gαi_3_-mGFP fusion proteins are insignificant. Therefore, only the configurations with Gα-mCitrine were investigated further [34]. For the activation of D_2_R, rotigotine hydrochloride was used as a full agonist, which exhibits an equally functional response as dopamine [43]. Rotigotine is not a selective dopamine D_2_R agonist, but in the present study, the cellular response induced by stimulation with this compound enabled us to observe the differences between the Gαi subtypes (Fig. 2)*.* It is noteworthy to mention that this agonist also binds efficiently to the dopamine D1-like receptors, which was confirmed by the measurements of cAMP levels. These results are in agreement with observations reported by other research groups [43–45].

The ability of rotigotine to activate D_2_R was confirmed by the measurements of intracellular cAMP levels taken in all the investigated settings (Fig. 2). However, data on the ability of this agonist to inhibit or activate adenylyl cyclase are limited in the literature. Most of the available studies focus on the thermodynamic and kinetic properties associated with binding to the receptor, or the overall pharmacological effect [46–48]. By contrast, in the present study, we analyzed the response of different subtypes of Gαi and Gαs by changes in the intracellular cAMP level following the stimulation of cells in which D_2_R and Gα were induced for overexpression. In the case of overexpression of only Gαi and D_2_R, two Gα subunits were able to inhibit adenylyl cyclase at a statistically significant level (Gαi_2_: *p* < 0.001; Gαi_3_: *p* < 0.05), except Gαi_1_. Additionally, as assumed, full Gαi heterotrimers showed an increased ability to interact with D_2_R, which was indicated by statistically significant differences when the effects of Gαi–D_2_R and Gαiβ_1_γ_2_–D_2_R interactions were compared. The higher level of Gβ_1_γ_2_ dimer present in cells supported the formation of the whole trimer more efficiently and influenced the behavior of the Gαi subunits investigated. It is worth noticing that in comparison with control conditions, the differences between the Gαi subunits diminished upon additional overexpression of Gβ_1_γ_2_ and only comparison between Gαi_1_β_1_γ_2_ and Gαi_2_β_1_γ_2_ showed significant differences in the cAMP level (*p* < 0.01); in the case of Gαi_3_β_1_γ_2_, such a phenomenon was not observed. The obtained results indicate that Gαi_2_ has the most profound influence on the inhibition of adenylyl cyclase, in the case of both overexpression of only Gα subunit or additional overexpression of Gβ_1_γ_2_ dimers. These results are in agreement with other studies, which used agonists such as dopamine, quinpirole, or N-n-propylnorapomorphine (NPA) [2, 27, 49, 50]. Moreover, incubation of cells overexpressing Gαs and D_2_R with rotigotine confirmed the ability of this agonist to induce the activation of adenylyl cyclase (*p* < 0.05). The ability to increase the cAMP level most probably results from the presence of other isoforms of adenylyl cyclase which are activated by the Gβγ dimer [51].

### Nanoscale distribution of Gαi and D_2_R monitored by FLIM–FRET and FRAP in living HEK293 cells

In the present study, we have shown that the FLIM–FRET and FRAP assays can be used to characterize the nanoscale spatial distribution of the closely related Gαi subunits in the plasma membrane, which in turn helped in assessing their role in the regulation of coupling preferences of D_2_R–Gαi proteins. Because of the unique spatial sensitivity, FRET was applied to elucidate the organization of the Gαi subunits and their heterotrimers formed with Gβ_1_γ_2_ in the plasma membrane. On the other hand, the FRAP technique was used to study the lateral dynamics of the investigated proteins in the cellular membrane. FRET was analyzed between the mCitrine-labeled Gαi or Gαs subunits (energy donor) and the mCherry-labeled D_2_R (energy acceptor). The emission spectrum of mCitrine was shown to overlap with the excitation spectrum of mCherry, making them a suitable donor–acceptor pair for FRET [52]. Following the energy transfer between the donor and the acceptor, the lifetime of mCitrine is shortened. This reduction in the fluorescence lifetime of the donor reflects the molecular proximity between the proteins that are linked to the fluorophores of the donor and acceptor. Thus, the FLIM–FRET technology allowed studying the membrane trafficking of Gαi as monomers and heterotrimers when co-expressed with D_2_R.

**Fig. 3:**
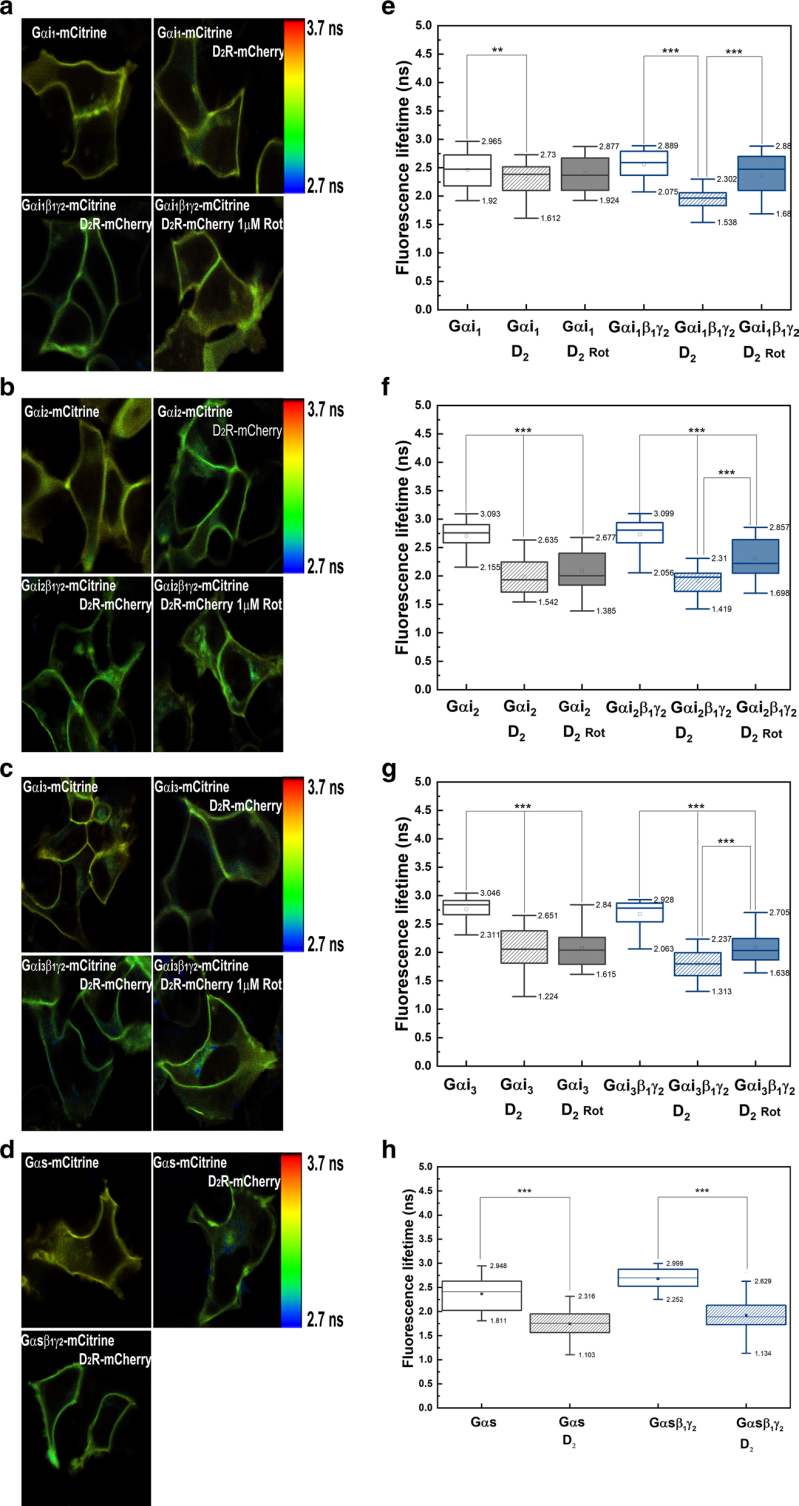
FLIM–FRET results. HEK293 cells were transiently transfected with Gα-mCitrine alone or both D_2_R-mCherry and Gα-mCitrine (donor and acceptor) with or without Gβ_1_γ_2_, or the donor in the presence of the acceptor after treatment with 1 μM rotigotine; mCitrine lifetime was measured: **a** Gαi_1_-mCitrine; **b** Gαi_2_-mCitrine; **c** Gαi_3_-mCitrine; **d** Gαs-mCitrine. Fluorescence lifetimes are presented in a continuous pseudo-color scale representing the time values ranging from 2.7 (blue) to 3.7 ns (red). **e**–**h** Box-and-whisker plots of the fluorescence lifetime *τ*_*1*_ of energy donor (Gα-mCitrine) and donor in the presence of acceptor (D_2_R-mCherry) are provided. The median is shown as a line in the box, while the bottom and top boundaries represent the lower and upper quartile, respectively. Statistical significance of the difference in the fluorescence lifetimes of the donor (*τ*_*1*_) was detected in the absence and presence of the energy acceptor using Mann–Whitney *U* test (**p < 0.005, ***p < 0.0001). Gαi_1_: n = 58; Gαi_1_ and Gβ_1_γ_2_: n = 40; Gαi_1_ and D_2_R with rotigotine: n = 34, without rotigotine: n = 61; Gαi_1_ and Gβ_1_γ_2_ and D_2_R with rotigotine: n = 26, without rotigotine: n = 58; Gαi_2_: n = 49; Gαi_2_ and Gβ_1_γ_2_: n = 44; Gαi_2_ and D_2_R with rotigotine: n = 41, without rotigotine: n = 47; Gαi_2_ and Gβ_1_γ_2_ and D_2_R with rotigotine: n = 31, without rotigotine: n = 46; Gαi_3_: n = 50; Gαi_3_ and Gβ_1_γ_2_: n = 54; Gαi_3_ and D_2_R with rotigotine: n = 33, without rotigotine: n = 68; Gαi_3_ and Gβ_1_γ_2_ and D_2_R with rotigotine: n = 33, without rotigotine: n = 79; Gαs: n = 39; Gαs and Gβ_1_γ_2_: n = 39; Gαs and D_2_R: n = 55; Gαs and Gβ_1_γ_2_ and D_2_R: n = 48

**Fig. 4:**
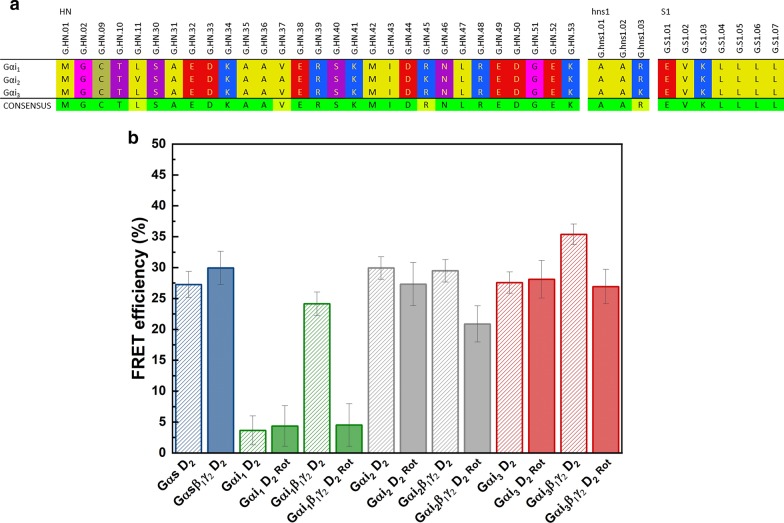
The difference in sequences of Gαi subunits and efficiency of energy transfer. **a** Multiple protein sequence alignment of human Gαi subunits encompassing residues of N-terminal fragment. The residues are colored to indicate their physicochemical properties: red—negatively charged amino acids, blue—residues with a positive charge, magenta indicates hydrogen bonding, and yellow shows hydrophobic aliphatic residues. In the consensus line, the positions in which there are differences in the sequence shown in yellow. Modified from gpcrdb.org (83). **b** A plot of calculated FRET efficiency percentage E derived from τ_1_; error bars represent standard errors

The fluorescence lifetime histograms obtained for mCitrine (Gαi_1_-mCitrine, Gαi_2_-mCitrine, Gαi_3_-mCitrine, Gαs-mCitrine) were fitted with a double-exponential decay function, and FLIM images showing the apparent lifetime of each pixel were generated. These images and the distribution of lifetimes with and without an acceptor in the HEK293 cells, which was estimated using SymPhoTime software, are shown in Fig. 3a–d. The FLIM images of the cells cotransfected with Gαi-mCitrine and D_2_R-mCherry showed a reduction in the apparent lifetime of the donor (change in color toward the blue hues across all pixels), compared to those expressing only Gαi-mCitrine. For example, the fluorescence lifetimes of mCitrine in the cells expressing Gαi_2_-mCitrine were estimated to be 2.76 ± 0.04 ns (*τ*_*1*_) and 3.23 ± 0.03 ns (*τ*_*2*_), with the amplitude of each of these lifetimes being approximately 40% and 60%, respectively. In the FRET system (cells additionally expressing D_2_R-mCherry), the donor emission curves were also fitted with the double-exponential decay model. However, shortening of the fluorescence lifetime that can be attributed to FRET was observed only in the case of the short component *τ*_*1*_, while the other component (*τ*_*2*_) remained almost unchanged. This indicates the involvement of only one donor species characterized by the lifetime *τ*_*1*_ in the energy transfer (FRETing donor state). Therefore, only the FRETing component was taken into account while calculating the FRET efficiency (Fig. 4b).

In the case of cells expressing Gαi_2_-mCitrine or Gαi_3_-mCitrine, cotransfection with D_2_R-mCherry significantly reduced the apparent lifetime of mCitrine to 1.93 ± 0.03 or 2.06 ± 0.04 ns, respectively (Fig. 3f, g). By contrast, the lifetime of Gαi_1_-mCitrine was decreased only slightly (2.39 ± 0.03 ns) in comparison to the *τ*_*1*_ estimated for the donor alone (2.48 ± 0.04 ns). The efficiencies of energy transfer between different Gαi subunits and D_2_R (Fig. 4b) calculated in the present study indicate that the spatial distribution of even closely related Gαi subunits differs. Here, we used Gαs subunit (not interacting with D_2_R) as a control, which has been reported to prefer localizing in the membrane region that differs from Gαi in lipid composition [53]; however, a significant FRET signal with D_2_R was also detected in this case (Fig. 4b). Combining these data, it can be concluded that dopamine D_2_R can exist in different membrane locations.

The apparent diffusion coefficients of monomeric subunits calculated in the study are summarized in Table 1. The lateral mobility of Gαi_1_-mGFP and Gαi_3_-mGFP was found to be similar (0.316 ± 0.012 and 0.338 ± 0.022 µm^2^ s^−1^, respectively). By contrast, the apparent diffusion coefficient of the Gαi_2_-mGFP subunit was much higher (0.474 ± 0.015 µm^2^ s^−1^). Interestingly, the diffusion characteristics of both Gαi_2_-mGFP and Gαi_3_-mGFP were not found to change significantly in the presence of dopamine D_2_R-mCherry at the cell membrane. However, for the Gαi_1_-mGFP subunit, mobility slightly increased (0.356 ± 0.014 µm^2^ s^−1^). This may have resulted from the competition between the receptor and the Gαi_1_-mGFP subunit for the membrane regions [36], which was confirmed by the results of the FRET experiments, with the poor efficiency of FRET between Gαi_1_-mGFP and D_2_R-mCherry indicating separate membrane localization.

**Table 1:** Lateral diffusion characteristics of Gα subunits in HEK293 cells in the presence of Gβ_1_γ_2_ and/or dopamine D_2_ receptor

	*D*_*app*_ (µm^2^ s^−1^)	*M*_*f*_ (%)	N
Gαs^a^	0.130 ± 0.004	84.5 ± 1.5	49
Gαs Gβ_1_γ_2_^b^	0.246 ± 0.009	92.4 ± 0.8	143
Gαs D_2_R	0.232 ± 0.009	89.0 ± 1.0	88
Gαsβ_1_γ_2_ D_2_R	0.245 ± 0.009	90.6 ± 1.1	110
Gαi_1_	0.316 ± 0.012	91.8 ± 1.4	55
Gαi_1_ Gβ_1_γ_2_	0.237 ± 0.010	88.2 ± 1.6	53
Gαi_1_ D_2_R	0.356 ± 0.014	92.2 ± 1.4	45
Gαi_1_β_1_γ_2_ D_2_R	0.291 ± 0.012	91.0 ± 1.2	60
Gαi_2_	0.474 ± 0.015	95.2 ± 0.7	120
Gαi_2_ Gβ_1_γ_2_	0.526 ± 0.019	94.1 ± 1.3	50
Gαi_2_ D_2_R	0.467 ± 0.020	93.1 ± 1.4	58
Gαi_2_β_1_γ_2_ D_2_R	0.345 ± 0.013	90.9 ± 1.3	60
Gαi_3_^a^	0.338 ± 0.022	94.2 ± 1.7	34
Gαi_3_ Gβ_1_γ_2_^b^	0.424 ± 0.014	93.5 ± 0.9	66
Gαi_3_ D_2_R	0.358 ± 0.016	90.1 ± 1.5	60
Gαi_3_β_1_γ_2_ D_2_R	0.381 ± 0.014	91.0 ± 1.4	50

In the experiments where the co-expression took place, the diffusion of Gα subunits was measured. Values represent the mean ± S.E.M

*D*_*app*_—apparent diffusion coefficient, *M*_*f*_—mobile fraction

^a^Data from Ref. [40]

^b^Data from Ref. [34]

The activation of D_2_R with rotigotine did not influence the FRET signal observed between the monomeric Gα and the agonist-receptor complex. This suggests that even if heterotrimers were formed between Gαi-mCitrine and endogenous Gβγ dimers, these complexes had no influence on the measured FRET. On the contrary, in the intracellular cAMP assay, a reduction in the level of cAMP was observed for all Gαi monomers (Fig. 2). This might have been caused, at least partly, by the assembly of an additional heterotrimer complex of Gαi-mCitrine fusion proteins with an endogenous Gβγ dimer. However, most cells showed an almost unchanged FRET signal upon receptor activation, which indicates that the concentration of heterotrimers formed with endogenous Gβγ dimer remains relatively low. In cells expressing additional Gβ_1_γ_2_, D_2_R activation resulted in a significant reduction in the FRET efficiency (Fig. 4b).

Thus, the data obtained from the FLIM–FRET and FRAP assays provide new insights about the location of the closely related Gαi subunits in the plasma membrane: (i) FRAP analysis of fluorescently labeled Gαi_2_ showed its significantly faster lateral diffusion compared to that of Gαi_3_ and Gαi_1_; (ii) FRET of fluorescently labeled D_2_R with Gαi_2_ and Gαi_3_ in the plasma membrane was higher than that with Gαi_1_. These together suggest the different distributions of Gαi subunits in the plasma membrane.

### Mapping the organization of Gαi heterotrimers in the plasma membrane

FRAP analysis was also performed for all the investigated subunits in the heterotrimeric system (Table 1). For this purpose, the HEK293 cells were cotransfected with additional vectors encoding Gβ_1_ and Gγ_2_ subunits to provide an excess of Gβγ dimers. As shown earlier, the Gβγ dimer was found to modulate the lateral diffusion of a heterotrimeric G protein compared to a monomeric Gα subunit [34]. In addition, the apparent diffusion coefficients of Gαi_2_ and Gαi_3_ with Gβ_1_γ_2_ complexes were estimated to be substantially higher compared to that of monomers (0.526 ± 0.019 and 0.424 ± 0.014 µm^2^ s^−1^, respectively). However, the presence of the Gβ_1_γ_2_ dimer had the opposite effect on the mobility of Gαi_1_ subunit, and the *D*_*app*_ value was found to decrease to 0.237 ± 0.010 µm^2^ s^−1^. This observed effect is particularly interesting in the case of Gαi_1_ and Gαi_3_ subunits because, as monomers with a high sequence identity, these subunits were characterized by identical lateral mobility.

For heterotrimeric Gαiβ_1_γ_2_ complexes, the presence of dopamine D_2_R in the cell membrane caused a significant change in the lateral mobility of all the investigated subunits. The biggest difference was observed for the Gαi_2_ subunit, for which the *D*_*app*_ value declined to 0.345 ± 0.013 µm^2^ s^−1^. Such a change in lateral diffusion may have resulted from an effective limitation of mobility to the receptor signaling platform areas such as the raft domains of the plasma membrane [54]. Although, for the Gαi_1_β_1_γ_2_ heterotrimer, a slight increase in *D*_*app*_ was observed (0.291 ± 0.012 µm^2^ s^−1^) in the presence of D_2_R, whereas the mobility of Gαi_3_β_1_γ_2_ slightly decreased in the presence of D_2_R compared to the heterotrimer alone (0.381 ± 0.014 µm^2^ s^−1^). However, it should be noted that in the presence of D_2_R the differences in the lateral diffusion rates of the investigated Gαi heterotrimers diminished. This might be due to the colocalization of all heterotrimers with D_2_R, which was proved by the FRET measurements.

As shown in Fig. 3e, g, the association of Gαi_1_ and Gαi_3_ with Gβ_1_γ_2_ dimers caused a further reduction in the apparent fluorescence lifetime of mCitrine in the presence of mCherry-fused D_2_R (box chart), and thus, there was an increase in the FRET efficiencies. The highest FRET efficiency of 35.4 ± 1.6% was detected in the cells co-expressing D_2_R and Gαi_3_β_1_γ_2_, while the lowest efficiency of 11% was found for the Gαi_1_β_1_γ_2_ heterotrimer. By contrast, Gαi_2_β_1_γ_2_ exhibited the same FRET signal with D_2_R as the monomeric Gαi_2_. However, the FRET efficiency of this subunit was found to be relatively high. The lifetime of Gαi_2_-mCitrine in the heterotrimeric complex was estimated as 2.0 ± 0.03 ns, which amounted to an energy transfer efficiency of 29.5 ± 1.8% (versus 29.9 ± 1.8% with monomeric Gαi_2_). These different patterns of changes in the FRET efficiency further point toward the difference in the membrane distribution of distinct Gαi subunits and D_2_R. Interestingly, in appropriate FRET pairs, differences were observed in the contribution of the fluorescence decay times for Gαi-mCitrine in heterotrimeric complex and the monomeric Gαi. The amplitude of the FRETing component (*τ*_*1*_) decreased to 17% and 25% for Gαi_1_β_1_γ_2_ and Gαi_3_β_1_γ_2_, respectively, whereas it remained at the same level for Gαi_2_β_1_γ_2_, similar to that calculated for the D_2_R–Gαi_2_ pair. The simplest explanation that could be provided for this effect is that the subpopulations of Gαi_1_ and Gαi_3_, for which the energy transfer to D_2_R-mCherry was reduced, relocate within the membrane upon the formation of the heterotrimeric complex. Thus, based on the FLIM data, it can be concluded that the receptor and heterotrimeric G protein in the basal state (before receptor activation) localize at the cell membrane within the same area (signaling platform), promoting signal transduction.

FRET analysis revealed that all the Gαi heterotrimers responded to the agonist rotigotine. The activation of D_2_R with rotigotine caused a significant reduction in the FRET efficiency in the case of all heterotrimers (Fig. 4b). The most pronounced decrease was observed for Gαi_1_β_1_γ_2_, while the lowest decrease was noted for Gαi_2_β_1_γ_2_. However, the difference observed in the FRET signal between the G protein and D_2_R before and after receptor stimulation cannot be interpreted as the magnitude of the rotigotine effect. D_2_R activation by agonist leads to the activation of G protein, which is accompanied by the dissociation of Gα from the Gβγ dimer, followed by Gα translocation within the plasma membrane and the internalization of the receptor. Because the complexes formed between the agonist-bound D_2_R and the G proteins have short lifetimes [55], which are off the time scale of the FLIM measurement, the recorded FLIM signal comes dominantly from the further steps of the signaling cascade. Thus, the FRET signal obtained after receptor stimulation should be compared to the value of FRET between monomeric Gαi and D_2_R. According to which the most pronounced effect of rotigotine was observed for Gαi_2_β_1_γ_2_.

## Discussion

Dopamine is a neurotransmitter that plays a critical role in controlling movement, cognition, and emotion. Dopamine receptors are expressed in neurons of the nigrostriatal pathway (motor-related), the mesolimbic-cortical pathway (reward system, emotional control) and tuberoinfundibular system [56]. Peripheral dopamine neurons are involved in renal and cardiovascular functions, and immune regulation. Dysfunction of dopaminergic pathways play an essential role in the pathophysiology of Parkinson's disease, schizophrenia, mood disorders, attention-deficit disorder, Huntington’s disease, Tourette's syndrome, Tardive dyskinesia, and other disorders. Therefore, insight into the selectivity of signal transduction between the dopamine receptor and G proteins is crucial for understanding of current therapies and development of new treatments. Interestingly, 21.9% of all GPCRs couple exclusively to the Gαi/o subfamily, another 5% couple to Gαi/o and of other G subfamilies [57]. All these receptors may couple differentially among various Gαi and Gαo isoforms, and individually prefer one specific isoform to the others [58–60].

The structural details behind the selectivity of receptor–G protein activation remain unclear and are an important subject of biochemical and biophysical studies [17, 23, 61, 62]. Studies dealing with the Gαs, Gαi, and Gαq families have shown their direct roles in regulating the levels of the secondary messenger and have provided substantial insight into the GPCR–G protein interface [63–65]. Despite that there is a plethora of data regarding the coupling specificity of various GPCRs, only a little is known about the potential receptor selectivity between the closely related members of the G protein families. In the present study, we have focused on the functional selectivity of dopamine D_2_R toward the Gα_i1_, Gα_i2_, and Gα_i3_ subunits, and analyzed whether the organization of Gαi heterotrimers in the plasma membrane can influence D_2_R signaling. This is particularly interesting in light of the current understanding of the complexity observed with the structural and functional organization of the cell membranes. The different lipid species present in membranes influence their properties, including the formation of membrane domains, as well as induce changes in the activity and density of the membrane proteins. However, it is not known whether the organization of G proteins in the plasma membrane influences their coupling with D_2_R or whether it might be one of the determinants of their coupling selectivity. Since it has been reported that there are differences in the plasma membrane targeting and trafficking pathways of the G proteins composed of Gα subunits belonging to different subfamilies, it is, therefore, reasonable to also evaluate the behavior of heterotrimers composed of closely related Gαi in the membranes, especially taking into account the already existing data [34, 53, 66, 67].

The membrane-binding area of Gα is limited to two sites on the surface of the protein and the membrane [67]. Its most critical membrane-binding determinant is the lipid anchors in conjunction with a polybasic motif at the N-terminus [66, 68, 69]. Depending on the specific subclass, the Gα subunits are palmitoylated and mostly myristoylated [70, 71]. All the Gαi subunits are N-myristoylated and S-palmitoylated, and the amino acid identity among them is high: Gαi_1_ and Gαi_3_ share a sequence identity of 94%, whereas Gαi_2_ has a lower identity of 87.5% and 85.5% to Gαi_1_ and Gαi_3_, respectively. We found two differences between the Gαi subunits in the positively charged motif at the N-terminus, which appear to be relevant (Fig. 4a). The first one concerns the position 21 where an R residue is present in Gαi_3_ and Gαi_1_, while a K residue is present in Gαi_2_. An additional substitution is found at position 32 of Gαi_3_, where K is present in the place of R in Gαi_1_ and Gαi_2_. However, when comparing the diffusion coefficients of Gαi_3_ and Gαi_1_, this position seems to be of lower importance in attaching Gαi to the membrane. Even if these substitutions did not appear to be significant, as they had no effect on the charge of the amino acid residues, it was assumed that they affect the interactions of Gαi with the membrane and might also influence the efficiency of translocation of Gα within the membrane. Our diffusion data suggest that the N-terminal residues of the Gα function as an essential signal to ensure the correct localization of the Gαi subunits at the plasma membrane.

The sequence differences in the polybasic motif between the Gαi subunits seemed to correlate well with the differences in their lateral diffusion coefficients detected by the FRAP experiments. The diffusion coefficient of the subunits increases in the following order: Gαi_1_ ≤ Gαi_3_ < Gαi_2_. Our data strongly suggest that the presence of the cluster of positively charged amino acids in the N-terminus of Gαi contributes to the membrane targeting of Gα, thus strengthening its affinity to the plasma membrane. The reduced membrane mobility of Gαi_1_ corresponds to the presence of a larger number of R residues in the polybasic motif. Both K and R function as basic residues; however, they differ in their geometric structure and possible interactions. Compared to the K residue, the R residue forms a higher number of electrostatic interactions, such as salt-bridges and hydrogen bonds, so it presumably results in stronger interactions than those generated by the lysine residue [72, 73]. The interactions that are observed for the positively charged residues include hydrogen bonds to the phosphate groups of phospholipids and electrostatic interactions to the negatively charged lipids at the cytosolic surface of the membrane [74]. Together, these interactions might cause retention of the positively charged residues on the cytoplasmic face of the membrane, slowing down the membrane mobility of the protein. In line with this hypothesis, in the present study, the Gαs subunit, which possesses a higher number of positively charged residues in the polybasic motif, showed the slowest membrane mobility among all the investigated Gα subunits.

On the other hand, the slower rate of lateral diffusion observed for Gα indicates that molecular motion is transiently confined, and such a protein resides within a particular region for a longer period of time. This in turn could enhance the FRET signal—in this case, the energy transfer between D_2_R and the slowest diffusing Gαi subunit (assuming a similar distribution for all the Gαi subunits across the membrane). As mentioned above, the FRET technique was applied to assess the trafficking of Gαi as monomers and heterotrimers when co-expressed with dopamine D_2_R and to analyze the corresponding changes in their relative membrane localization. Interestingly, we detected that the resonance energy transfer between D_2_R and the slowest diffusing Gαi subunit—Gαi_1_—had the lowest efficiency (almost none). The highest FRET signal was observed for the fastest diffusing Gαi_2_, while a slightly less efficient signal was recorded for Gαi_3_ (diffusion rates comparable to Gαi_1_) and Gαs (the slowest diffusion rate). These results indicate that the sequestration of Gα subunits, even those belonging to the same Gαi subfamily, in the plasma membrane may also vary.

In our earlier work, which investigated the distribution of Gαs and Gαi_3_ in the plasma membrane, we observed that these proteins were localized in different types of specific membrane domains [53]. It was found that the Gαs subunits preferred solid-like domains (insensitive to cholesterol, with a structure or composition of lipid rafts), while the Gαi_3_ subunit preferred the more fluid regions of the membrane and detergent-resistant domains such as lipid rafts. This suggests that distinct protein acylation may act as a signal for recruitment or retention into particular membrane regions/domains containing specific lipids. As already mentioned, despite that all the Gαi subunits had the same lipid anchors, the difference in the sequence of the polybasic region (for example, the presence of additional R residues) has an impact on the lipid preference and membrane localization of the subunits. A similar value of apparent diffusion coefficient observed for Gαi_1_ and Gαi_3_ in this study suggests their similar membrane localization. Therefore, it is tempting to conclude that Gαi_1_ also prefers detergent-resistant and cholesterol-dependent membrane domains (i.e. Lo-like domains) in the plasma membrane. However, the FRET data do not support this interpretation (as different FRET efficiency was estimated for the pairs D_2_R–Gαi_1_ and D_2_R–Gαi_3_). The main limitation of the FRAP studies is that it does not provide detailed information about where the species are present and the subpopulations in different locations cannot be simply identified. Another important aspect that needs to be considered is the nature of the interaction of the R residue with the membrane, as in some cases it leads to a local distortion of the bilayer around proteins [74]. This distortion is manifested in a high level of local water penetration inside the membrane, and can lead to a decrease in the thickness of the bilayer as well as affect the long-range interactions. We cannot rule out that the lower FRET signal observed between D_2_R and Gαi_1_ could result from the local deformation of the membrane induced by Gαi_1_, which affects the distribution and density of D_2_R in such an area in the membrane. This scenario is quite probable since the N-terminus of Gαi_1_ is arginine-rich, and the dopamine D_2_R is broadly distributed throughout the cell membrane, as supported by our FRET results. It has been shown by Sharma et al. that D_2_R exists in both detergent-soluble and detergent-insoluble fractions of the plasma membrane [36]. The similar plasma membrane localization of the Gαi_1_ and Gαi_3_ subunits cannot be excluded; however, faster diffusion of Gαi_2_ indicates that this subunit is localized within the membrane area which is composed of different types of lipids, a more fluid membrane area, and rich in D_2_R, as suggested by our FRET data.

As reported in our previous work, the diffusion of the Gαs and Gαi_3_ subunits speeds up upon the formation of heterotrimer [34, 40]. The Gβγ dimer is responsible for the rapid relocation of Gα from the lamellar membrane region where it resides as a monomer [67]. As expected, the membrane mobility of Gαi_2_β_1_γ_2_ and Gαi_3_β_1_γ_2_ changed in a similar way. The association of Gβ_1_γ_2_ with the GDP-bound Gαi_2_ or Gαi_3_ caused the G proteins to relocate into the more fluid membrane regions (the diffusion rate increased despite the increase in the molecular weight of the complex). However, an opposite behavior was observed for Gαi_1_. In the case of this subunit, when the trimer was formed, its membrane mobility slowed down the diffusion rate. This implies that—in contrast to Gαi_2_ and Gαi_3_—Gαi_1_ in the heterotrimer complex did not change its lipid environment or changed it only slightly (the formation of heterotrimers by Gαi_1_ did not involve the translocation of this protein into a more fluid region in the membrane as noted for the rest of the Gαi subunits). Alternatively, the observed slow-down of the heterotrimer diffusion could be a sustained effect of the structural perturbations of the lipid bilayers that were caused by the N-terminus of Gαi_1_, which also affected the diffusion of the full heterotrimeric complex. To clearly discriminate between these possibilities, structural in vitro studies in a model system (purified proteins and lipid bilayers) are required. However, the impact of the Gβ_1_γ_2_ dimer on the membrane distribution of the complete complex of Gαi_1_β_1_γ_2_ is unquestionable, as was indicated by the significant FRET signal between the complete heterotrimer and D_2_R compared to the almost undetectable signal of the monomeric Gαi_1_. Previous studies of fluorescence and electron paramagnetic resonance have also pointed out that the N-terminus of Gαi_1_ undergoes a conformational change upon Gβγ binding and activation [75].

Taken together, this new experimental evidence strengthens our earlier hypothesis that the Gβγ dimer alone does not define the affinity or specificity of the complete heterotrimer toward the membrane lipid phase [34]. In general, the distinct heterotrimeric combinations showed differences in their mobility characteristics (Table 1). Therefore, the interplay between the Gβγ and Gα subunits is critical for controlling the trafficking of the complete G protein heterotrimer. Moreover, we found that for some heterotrimers, Gα acts as a crucial modulator of the membrane localization. These findings appear to be in contradiction with the previously published results of the nuclear magnetic resonance-based studies (on purified proteins and liposomes), suggesting that only Gβγ dimer is responsible for the cellular localization of the heterotrimeric Gαi_1_ proteins, thereby masking the lamellar membrane affinity of Gαi_1_ [67].

Besides the observed changes in diffusion rates, the Gβ_1_γ_2_-dependent translocation of the Gαi_1_ and Gαi_3_ subunits also induced an increase in the FRET efficiency. The simplest explanation that can be given for this phenomenon is that the heterotrimers are localized within the D_2_R-rich membrane fraction and are waiting for the agonist-activated receptor. By contrast, we found that the FRET signal between Gαi_2_β_1_γ_2_ and D_2_R remained at the same level as that calculated for the D_2_R–Gαi_2_ pair. Since the monomeric Gαi_2_ is located in the D_2_R-rich membrane region, it is most likely that its spatial distribution undergoes only a slight change upon heterotrimer formation. Our data are in general agreement with the results published by Sharma et al. [36] who observed that the majority of the plasma membrane-expressed population of D_2_R was located within the detergent-resistant structures that do not correspond to classical lipid rafts. Treatment with an agonist led to the loss of both the detergent-soluble and detergent-resistant D_2_R fractions; however, the loss of detergent-resistant fraction was significantly greater.

In the present study, we found that the use of an antiparkinsonian drug, rotigotine, as a D_2_R agonist led to the inhibition of cAMP production and noticed a difference in the coupling selectivity of heterotrimers. The order of rotigotine potency (Gαi_2_ > Gαi_3_ = Gαi_1_) observed in the FRET experiments remains in general agreement with the results shown by direct measurement of the level of intracellular cAMP. Because the complexes formed between agonist-bound D_2_R and G proteins have short lives, the time-resolution of the FRET measurements allowed detecting only the further steps in the signaling cascade: dissociation of Gα from the trimer complex, followed by its relocation within the plasma membrane and receptor internalization. All these processes together were manifested as a decrease in the FRET efficiency, as compared to the basal signal (FRET for D_2_R–Gαi pair). It is noteworthy that in several earlier reports, Gαi_2_ was also indicated as selective toward D_2_R, causing maximal inhibition of adenylate cyclase [26, 76, 77]. However, the experimental data imply that the coupling selectivity of Gαi is regulated by the agonist-activated conformation of D_2_R. For example, stimulation of D_2_R with R(+)-3-PPP hydrochloride caused preferential coupling to Gαi_3_ rather than Gαi_1_ or Gαi_2_ [25]. The C-terminal Gα, as well as the movement magnitude of the sixth transmembrane helix of activated receptor, which varies from one receptor to another, has been predicted to be the main modulator of the selectivity of the G protein subtypes. Regarding the Gαi subunits, the sequence of C-terminal helix is almost identical for all proteins (two substitutions in Gαi_3_: 350D/E, 354F/Y). Furthermore, neither the amino acid sequences of β2–β3 loop nor the β6 sheet in the Ras-like domain—additional residues predicted as selectivity determinants—show any significant differences (one substitution in Gαi_3_, 195H/Y) [15, 23]. This clearly indicates that there must be other selectivity determinants for the coupling of Gαi heterotrimers. Our data support the new idea that membrane location can serve as an important selectivity determinant of downstream signaling. Considering that the ligated receptors might be clustered [78, 79] for longer durations within the given domains in the cell membrane, which also contain appropriate G protein, it seems likely that this combination fine-tunes the sensitivity and specificity of a given signaling pathway.

## Conclusions

The concept of rapid translocation of the Gα monomers after dissociation from the Gβγ dimer and their localization to the lamellar structures, where they interact with effector molecules, is widely accepted and has also been confirmed by numerous studies [6, 80, 81]. The model of membrane localization-dependent signaling by G protein has been proposed over a decade ago. However, since then, the knowledge of membrane organization and functioning has significantly evolved [54, 82]. Therefore, some aspects of this model require revision. For instance, all the monomeric Gαi subunits are considered as identical in terms of their membrane coupling, and it has been postulated that they localize to the same type of membrane structures—lipid raft domains [31, 32]. In fact, as proved in the present study, even closely related subunits of Gαi differ in their membrane trafficking properties that influence their signaling. The interactions between lipids and proteins seem to be crucial in GPCR-dependent cell signaling due to their functional organization in specific membrane domains, and should therefore be taken into account as one of the selectivity determinants of G protein coupling.

## Supplementary information

**Additional file 1**. Supplemental RT-qPCR experiments.

## Data Availability

The datasets generated during and/or analysed during the current study are available from the corresponding author on reasonable request.

## Abbreviations

GPCR: G protein-coupled receptor
D_2_R: Dopamine D_2_ receptor
FRAP: Fluorescence recovery after photobleaching
FRET: Fluorescence resonance energy transfer
FLIM: Fluorescence-lifetime imaging microscopy
HEK293: Human embryonic kidney 293 cells
cAMP: 3′,5′-Cyclic adenosine monophosphate
GTP: Guanosine-5′-triphosphate
GDP: Guanosine diphosphate
ICL: Intracellular loops
TM: Transmembrane helix
RGS: Regulators of G protein signaling
AGS: Receptor-independent activators of G-protein
PCR: Polymerase chain reaction
mGFP: Monomeric Green Fluorescent Protein
FP: Fluorescent protein
MEM: Minimum essential medium
FBS: Fetal bovine serum
NPA: N-n-propylnorapomorphine
D_app_: Apparent diffusion coefficient
M_f_: Mobile fraction
