# Supplementary material for: The Gαi protein subclass selectivity to the dopamine D2 receptor is also decided by their location at the cell membrane
Source: Cell Commun Signal. 2020 Dec 11;18:189. doi: 10.1186/s12964-020-00685-9 (PMC7731117; doi:10.1186/s12964-020-00685-9)
Supplement: Supplementary file 2 — Additional file 1. Supplemental RT-qPCR experiments. [file 12964_2020_685_MOESM2_ESM.pdf]

Electronic supplementary material for:

"The Gαi protein subclass selectivity to the dopamine D_2 receptor is also decided by their location at the cell membrane"

Agnieszka Polit*, Beata Rysiewicz, Paweł Mystek, Ewa Błasiak, Marta Dziedzicka-Wasylewska

Department of Physical Biochemistry, Faculty of Biochemistry Biophysics and Biotechnology, Jagiellonian University, Gronostajowa 7, 30-387 Kraków, Poland

*Correspondence to: a.polit@uj.edu.pl; Tel.: +48 12 6646156

Beata Rysiewicz, e-mail: beata.rysiewicz@doctoral.uj.edu.pl

Paweł Mystek, e-mail: pawel.mystek@uj.edu.pl

Ewa Błasiak: e-mail: ewa.blasiak@uj.edu.pl

Marta Dziedzicka-Wasylewska, e-mail: marta.dziedzicka-wasylewska@uj.edu.pl

RT-qPCR

Cell cultures were prepared in the same manner as for microscopic studies. Total RNA was extracted using Trizol Reagent (Invitrogen). mRNA was converted to cDNA via RevertAid Reverse Transcriptase (Thermo Scientific) according to the manufacturer's instructions with Oligo(dT)$_{16}$ primers (Genomed). cDNA was then mixed with gene-specific primers (Table ESM1, Genomed) and Luminaris Hi Green qPCR Master Mix (Thermo Scientific) for PCR amplification using an Eco Real-Time PCR System (Illumina). Amplifications were carried out in 10 µl reaction solutions containing 5 µl Luminaris Hi Green, 1 µl cDNA and 0.3 µM of each specific primer pair. PCR conditions were 95°C for 10 min followed by 40 cycles of 95°C for 15 s, 60°C for 30s and 72°C for 30 s. The specificity of each pair of primers was checked by melting curve analysis (95°C for 15 s, 55°C for 15 s and continuous raise in temperature to 95°C at 1°C /3 s). Each assay was performed with technical duplicates. Transfections, RNA extraction, and RT-qPCR experiments were performed in three independent experiments. In order to check if specific primers were not compatible with other Gαi cDNAs, cross reactions were carried out.

The G protein mRNA expression was determined by analyzing the threshold cycle for each primer set in material from both transfected HEK293 cells and non-transfected HEK293 cells (control). The data were analyzed with the ΔΔCq method with the efficiency correction (Pfaffl method) and presented as fold change in gene expression normalized to an endogenous GAPDH gene and relative to the endogenous expression in non-transfected control.

Table ESM1. Pairs of primers used in qPCR reactions

Gene target	Forward primer	Reverse primer
$G\alpha i_1$	AAGCCTGTTTCAACAGATCCCGAGAG	CACTCTAGTTCTGAGAACATCTTGTTGAGTC
$G\alpha i_2$	CCGAGGAGGAATGCCGGCAG	CTCGGCGGTGCAGGACAGTG
$G\alpha i_3$	AGCAGGAGTGATTAAACGGTTATGGCG	GCTGAGTTGGAATGTAGTTAGACTGGGA
Gαs	CTGAGGATGCTACTCCCGAGCC	AGTAGTGACGCCCATCTCCACTGG
GAPDH	CAACAGCGACACCCACTCCTCC	GGTCTTACTCCTTGGAGGCCATGTG

The relative expression of three different Gαi subunits coexpressed with the D_2R is comparable. There is no significant difference in the mRNA expression level in cells overexpressing Gα subunits and D_2R receptor and those overexpressing also $G\beta_1$ and $G\gamma_2$ subunits of G proteins. The fold change in Gαs mRNA level, used as a control protein in this study, is smaller in comparison to Gαi subunits because of the fact, that the endogenous expression level of this protein is higher (1).

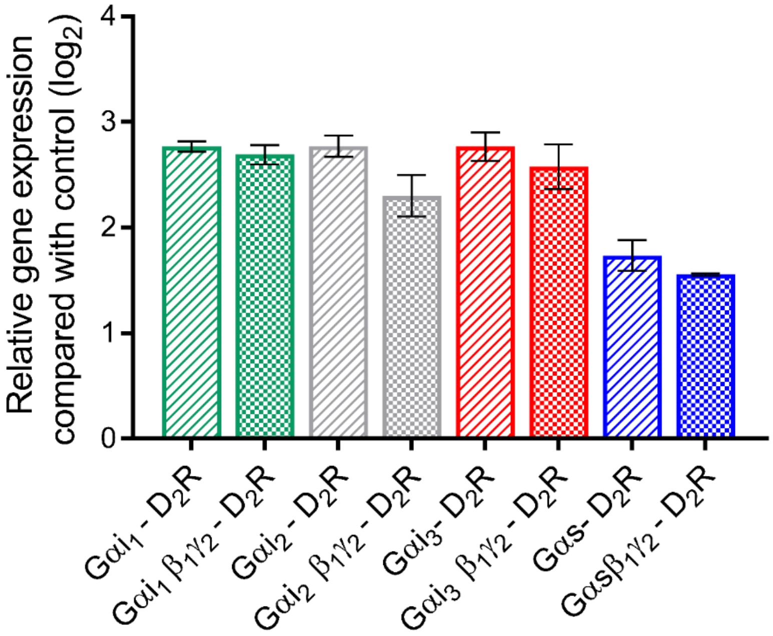

Figure ESM1. Relative gene expression. Total mRNA was extracted from HEK293 cells transiently transfected with Gα-mCitrine and D_2R-mCherry or Gα-mCitrine$\beta_1\gamma_2$ and D_2R-mCherry and used in RT-qPCR experiments. Data are presented as a logarithm of the relative gene expression compared with non-transfected cells. Error bars are SEM, unpaired *T-test* showed no significant differences.

1. Atwood BK, Lopez J, Wager-Miller J, Mackie K, Straiker A. Expression of G protein-coupled receptors and related proteins in HEK293, AtT20, BV2, and N18 cell lines as revealed by microarray analysis. BMC Genomics. 2011;12:14
